# HapX Mediates Iron Homeostasis in the Pathogenic Dermatophyte *Arthroderma benhamiae* but Is Dispensable for Virulence

**DOI:** 10.1371/journal.pone.0150701

**Published:** 2016-03-09

**Authors:** Antje Kröber, Kirstin Scherlach, Peter Hortschansky, Ekaterina Shelest, Peter Staib, Olaf Kniemeyer, Axel A. Brakhage

**Affiliations:** 1 Junior Research Group Fundamental Molecular Biology of Pathogenic Fungi, Leibniz Institute for Natural Product Research and Infection Biology, Hans Knöll Institute (HKI), Jena, Germany; 2 Department of Molecular and Applied Microbiology, Leibniz Institute for Natural Product Research and Infection Biology (HKI), Jena, Germany; 3 Department of Biomolecular Chemistry, Leibniz Institute for Natural Product Research and Infection Biology (HKI), Jena, Germany; 4 Systems Biology and Bioinformatics, Leibniz Institute for Natural Product Research and Infection Biology (HKI), Jena, Germany; 5 Institute of Microbiology, Friedrich Schiller University, Jena, Germany; Universidade de Sao Paulo, BRAZIL

## Abstract

For many pathogenic fungi, siderophore-mediated iron acquisition is essential for virulence. The process of siderophore production and further mechanisms to adapt to iron limitation are strictly controlled in fungi to maintain iron homeostasis. Here we demonstrate that the human pathogenic dermatophyte *Arthroderma benhamiae* produces the hydroxamate siderophores ferricrocin and ferrichrome C. Additionally, we show that the iron regulator HapX is crucial for the adaptation to iron starvation and iron excess, but is dispensable for virulence of *A*. *benhamiae*. Deletion of *hapX* caused downregulation of siderophore biosynthesis genes leading to a decreased production of siderophores during iron starvation. Furthermore, HapX was required for transcriptional repression of genes involved in iron-dependent pathways during iron-depleted conditions. Additionally, the Δ*hapX* mutant of *A*. *benhamiae* was sensitive to high-iron concentrations indicating that HapX also contributes to iron detoxification. In contrast to other pathogenic fungi, HapX of *A*. *benhamiae* was redundant for virulence and a Δ*hapX* mutant was still able to infect keratinized host tissues *in vitro*. Our findings underline the highly conserved role of the transcription factor HapX for maintaining iron homeostasis in ascomycetous fungi but, unlike in many other human and plant pathogenic fungi, HapX of *A*. *benhamiae* is not a virulence determinant.

## Introduction

The fungal pathogen *Arthroderma benhamiae* belongs to a group of fungi known as dermatophytes, which exclusively infect keratinized structures such as hair, skin (stratum corneum) and nails of humans and animals [1]. In recent years, an increasing number of *A*. *benhamiae* infections have been observed worldwide [2]. Amongst others, the main reservoir of the zoophilic species *A*. *benhamiae* is the guinea pig [3] and infections of humans often occur after direct contact with an animal carrying the fungus. Usually, the infections are superficial and not life-threatening, but even in immunocompetent hosts, dermatophytosis is long-lasting and difficult to cure [4]. To date, only few putative virulence factors of dermatophytes have been identified and investigated at the molecular level yet. These are, for example, the ABC transporter TruMDR2 and pH signalling transcription factor PacC of *Trichophyton rubrum* as well as the keratinolytic proteases Sub3 of *Microsporum canis* and Sub6 of *Trichophyton mentagrophytes* [5–10]. Recent advances in genetic manipulation of *A*. *benhamiae* have set a basis for fundamental genetic research of dermatophytes [11]. *A*. *benhamiae* has proven to be an ideal model organism because it grows relatively fast and allows efficient targeted gene deletion as well as gene complementation [12]. Additionally, the complete genome sequence and global transcriptional profiles are available, and comprehensive *in vitro* and *in vivo* infection models have been established [13–15].

Iron is an essential trace element for almost all organisms. Its ability to exist in two redox states makes iron an important cofactor of proteins involved in a variety of cellular processes, including respiration. On the other hand, iron excess is toxic because it catalyzes the production of cell-damaging hydroxyl radicals in the presence of oxygen [16]. Thus, cellular uptake, storage and utilization of iron need to be tightly regulated to avoid the formation of reactive oxygen species. In the filamentous fungi *Aspergillus nidulans* and *Aspergillus fumigatus*, iron homeostasis is regulated by the transcription factors HapX and SreA which are interconnected by a negative regulatory feedback loop [17–20]. The Cys_2_-Cys_2_-type GATA zinc finger transcription factor SreA downregulates the expression of *hapX* and other genes during iron sufficiency by binding to a specific motif within the promoter region. SreA represses siderophore biosynthesis and reductive iron assimilation to avoid iron excess during iron sufficiency [21]. The basic region leucine zipper (bZIP) transcription factor HapX downregulates the expression of *sreA* during iron starvation by protein-protein interaction with the heterotrimeric CCAAT-binding complex (CBC) and by sequence-specific DNA binding [22, 23]. During iron-depleted conditions, the CBC-HapX complex represses iron-consuming pathways, including heme biosynthesis, tricarboxylic acid cycle and respiration to spare iron. On the other hand, it activates reductive iron assimilation, siderophore biosynthesis and siderophore uptake for iron acquisition [18, 19, 24]. Additionally, HapX is essential for iron detoxification by activating the vacuolar iron importer CccA under high-iron conditions [22, 25]. Due to its central role in iron homeostasis, the transcription factor HapX has shown to be a virulence determinant in several human fungal pathogens, such as *A*. *fumigatus*, *Candida albicans*, *Cryptococcus neoformans* as well as in the plant pathogenic fungus *Fusarium oxysporum* [19, 26–29]. Remarkably, until now, the role of iron during the infection of keratinized host tissues by dermatophytes has not been elucidated.

In this study, we have set out to investigate the function of the transcription factor HapX in *A*. *benhamiae*. We demonstrate that HapX function is crucial for the adaptation to iron starvation and iron excess, but is dispensable for the infection of keratinized host tissue.

## Materials and Methods

### Strains, media and growth conditions

The wild-type strain *A*. *benhamiae* LAU2354-2 = CBS 112371 = IHEM 20161 [30] was used for the generation of deletion mutants and reconstituted strains. For short-term storage, the wild-type *A*. *benhamiae* LAU2354-2 was cultivated on Sabouraud glucose agar [1% (w/v) peptone, 2% (w/v) glucose, 1.5% (w/v) agar] (SAB) and transformants of *A*. *benhamiae* LAU2354-2 were grown on SAB supplemented with 200 μg/ml hygromycin (ForMedium, Hunstanton, UK) or G418 (Carl Roth, Karlsruhe, Germany), according to the selectable marker used. Additionally, all fungal strains used in this study were stored as frozen glycerol stocks at -80°C (Table 1). Production of microconidia was performed on MAT agar [0.1% (w/v) peptone, 0.2% (w/v) glucose, 0.1% (w/v) MgSO_4_, 0.1% (w/v) KH_2_PO_4_; Carl Roth, Karlsruhe, Germany] if not otherwise stated. Further experiments were carried out at 30°C in *Aspergillus* minimal medium (AMM) containing 1% (w/v) glucose as the carbon source and 20 mM glutamine as the nitrogen source [31]. For solid AMM, 1.5% (w/v) agar was added. For iron-depleted conditions, iron was omitted (-Fe). Iron-replete media was supplemented with 0.03 mM FeSO_4_ (+Fe). For harsh iron starvation conditions, the ferrous iron chelator bathophenanthroline disulfonic acid Na_2_-salt (BPS) (Serva, Heidelberg, Germany) was used at a final concentration of 0.2 mM (-Fe +BPS). As xenosiderophore, the ferric iron chelator deferoxamine mesylate salt (DFOM) (Sigma-Aldrich, Taufkirchen, Germany) was added to the medium at a final concentration of 10 μM (-Fe +DFOM). For growth inhibition assays, 10^4^ microconidia of *A*. *benhamiae* wild type, Δ*hapX* mutant and *hapX*^*C*^ reconstituted strain were spotted on solid AMM agar supplemented with iron concentrations ranging from 1–10 mM FeSO_4_.

**Table 1:** *A*. *benhamiae* strains used in this study.

Strain	Parent	Genotype	Reference
LAU2354-2		wild-type strain	[30]
AbenHAPXM1A	LAU2354-2	Δ*hapX*::P_*gpd*_-*hph*-T_*trpC*_	This study
AbenHAPXM1B	LAU2354-2	Δ*hapX*::P_*gpd*_-*hph*-T_*trpC*_	This study
AbenHAPXK1A	AbenHAPXM1A	Δ*hapX*::*hapX*-T_caACT1_-P_*ACT1*_-*neo*	This study
AbenHAPXK1B	AbenHAPXM1B	Δ*hapX*::*hapX*-T_caACT1_-P_*ACT1*_-*neo*	This study

### Plasmid construction

**Fig 1:**
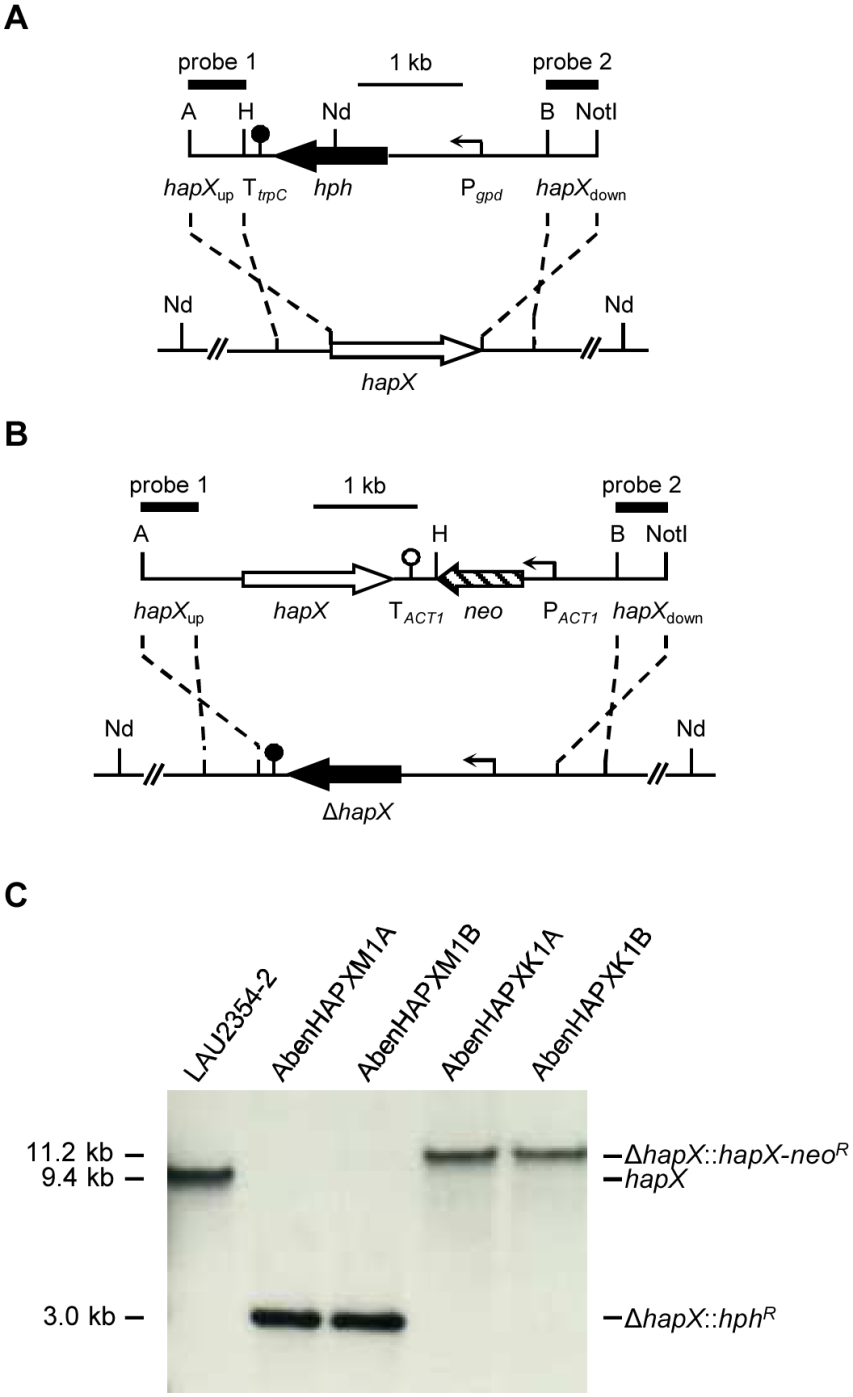
Generation of *A*. *benhamiae* Δ*hapX* mutants and reconstituted strains. (A) For deletion of the *hapX* locus (white arrow) in the wild-type strain *A*. *benhamiae* LAU2354-2 (bottom) a DNA cassette, containing the hygromycin resistance gene *hph* (black arrow) under control of the *gpd* promoter (P_*gpd*_, bent arrow) together with the termination sequence fragment T_*trpC*_ (filled circle) flanked by *hapX* upstream and downstream regions (*hapX*_up_ and *hapX*_down_, solid lines), was used (top). (B) For reinsertion of the *hapX* gene into its original locus in the Δ*hapX* mutants a DNA cassette, containing the coding region of *hapX* and the neomycin resistance gene *neo* (lined arrow) under control of the *A*. *benhamiae* actin promoter (P_*ACT1*_, bent arrow) together with the *Candida albicans* actin termination sequence fragment T_*ACT1*_ (blank circle) flanked by *hapX* upstream and downstream regions (*hapX*_up_ and *hapX*_down_, solid lines), was used. (C) Southern blot of *Nde*I-digested genomic DNA of the wild-type strain *A*. *benhamiae* LAU2354-2, Δ*hapX* mutants and *hapX*^*C*^ reconstituted strains with *hapX*-specific probe 1. The probes which were used for Southern analysis of the transformants are indicated by the black bars. Only the following relevant restriction sites are given in panels a and b: A, *Apa*I; B, *Bam*HI; H, *Hin*dIII; Nd, *Nde*I; *Not*I. The sizes of the hybridizing fragments are given on the left and their identities on the right.

Sequence information for the gene *hapX* (ARB_06811) was obtained from the annotated *A*. *benhamiae* genome [13]. Plasmid construction was performed as described before [12]. For the generation of the deletion mutants, up- and downstream sequences of the *hapX* gene were cloned successively in the plasmid pHPH1 [12]. For deletion of the entire coding region of *hapX*, an *Apa*I-*Hin*dIII fragment containing *hapX* upstream sequences from positions -518 to -4 with respect to the start codon was obtained by PCR with the primers AbenHAPX-1/AbenHAPX-2. Genomic DNA from the wild-type strain *A*. *benhamiae* LAU2354-2 was used as a template. A *Bam*HI-*Not*I fragment with *hapX* downstream sequences from positions +1417 to +1894 was amplified by PCR with the primers AbenHAPX-3/AbenHAPX-4. The *hapX* upstream and downstream sequences were successively cloned via the introduced restriction sites in plasmid pHPH1 to result in plasmids pAbenHAPXM1 and pAbenHAPXM2, respectively (Fig 1A). For reinsertion of the *hapX* gene into the knock-out mutant, the plasmid pAbenHAPXK2 was generated as follows. An *Apa*I-*Bgl*II DNA fragment including the *hapX* gene and *hapX* upstream sequences from positions -1008 to +1425 was amplified by PCR with the primers AbenHAPX-5/AbenHAPX-9. The *Bam*HI-*Not*I fragment with *hapX* downstream sequences from positions +1417 to +1894 (amplified with the primers AbenHAPX-3/AbenHAPX-4) was cloned in the *Bam*HI-*Not*I digested plasmid pNEO1 [12] yielding pAbenHAPXK1. The PCR product was cloned via the introduced *Apa*I and *Bgl*II restriction sites together with the *Bgl*II-*Hin*dIII [CaACT1T] fragment from pJetGFPACT1T1 [12] in the *Apa*I-*Hin*dIII digested plasmid pAbenHAPXK1 to give plasmid pAbenHAPXK2 (Fig 1B). All primers used for plasmid construction in this study are listed in S1 Table.

### Transformation of *A*. *benhamiae*

Transformation of *A*. *benhamiae* was carried out as previously described [12]. The Δ*hapX* mutant and the *hapX*^*C*^ reconstituted strain were generated by homologous recombination. Briefly, protoplasts produced from *A*. *benhamiae* microconidia were transformed with the constructed linear DNA cassettes from plasmids pAbenHAPXM2 (Δ*hapX* mutant) and pAbenHAPXK2 (*hapX*^*C*^ reconstituted strain). Hygromycin or neomycin resistant transformants were selected with either 250 μg/ml hygromycin or G418 depending on the selection marker used. For analysis of the transformants, fungal genomic DNA was isolated as stated before [12]. Targeted gene disruption or gene complementation was confirmed via Southern analyis using the Amersham ECL direct nucleic acid labeling and detection system (GE Healthcare, Little Chalfont, UK) according to the manufacturer’s instructions (Fig 1C).

### Determination of cell dry weight

A volume of 100 ml AMM was used for cell dry weight determination during iron-replete conditions (+Fe), iron limitation (-Fe), harsh iron starvation (-Fe +BPS) and in the presence of DFOM (-Fe +DFOM). For high iron concentrations 50 ml AMM was supplemented with 1 mM, 3 mM, 5 mM and 7 mM FeSO_4_. The medium was inoculated with 10^6^ microconidia per ml of the respective fungal strain and incubated at 30°C and 200 rpm. After 5 d of cultivation the mycelium was harvested by Miracloth (Calbiochem^®^, Merck Millipore, Darmstadt, Germany), thoroughly dried at 50°C and weighed.

### Identification of siderophores produced by *A*. *benhamiae*

For identification of siderophores produced by *A*. *benhamiae*, fungal cultures were grown in AMM without iron for 5 d at 30°C and 200 rpm. The mycelium was harvested by Miracloth and the culture supernatant was collected. The supernatant was exhaustively extracted with ethylacetate and the resulting extract dried with Na_2_SO_4_ and concentrated under reduced pressure. For HPLC analysis the dry extract was re-dissolved in 200 μL of methanol and filtered (Ultrafree filtration system for laboratory centrifuges, Oxy-Fill Rotrac^®^ membrane). The aqueous residue was freeze-dried and extracted with methanol. The extract was filtered using a paper filter, concentrated under reduced pressure and finally redissolved in 4 mL of 50% MeOH (H_2_O, v/v) for HPLC analysis. The fungal mycelium was freeze-dried, extracted with 10 mL of 50% MeOH (H_2_O, v/v) and filtered using a paper filter. Next, the filtrate was concentrated under reduced pressure and dissolved in 100 μL of 50% MeOH (H_2_O, v/v) for HPLC analysis. To convert the Fe-free derivatives into the iron complexes, all extracts were supplemented with 3 mM FeCl_3_ before HPLC analyses.

Analytical HPLC was performed on a Shimadzu LC-10Avp series HPLC system consisting of an autosampler, high-pressure pumps, column oven and PDA. HPLC conditions: C18 column (Eurospher 100–5, 250 x 4.6 mm) and gradient elution (MeCN/0.1% (v/v) TFA 0.5/99.5 in 30 min to MeCN/0.1% (v/v) TFA 100/0, MeCN 100% for 10 min), flow rate 1 mL min^−1^; injection volume: 30 μL. LC-MS measurements were performed using an Exactive Orbitrap High Performance Benchtop LC-MS with an electrospray ion source and an Accela HPLC system (Thermo Fisher Scientific, Bremen, Germany). HPLC conditions: C18 column (Betasil C18 3 μm 150 x 2.1 mm) and gradient elution (MeCN/0.1% (v/v) HCOOH (H_2_O) 5/95 for 1 min, going up to 98/2 in 15 min, then 98/2 for another 3 min; flow rate 0.2 mL min^−1^) or a Q Exactive Orbitrap High Performance Benchtop LC-MS with an electrospray ion source and an Accela HPLC system (Thermo Fisher Scientific, Bremen, Germany). HPLC conditions: C18 column (Accucore C18 3 μm 100 x 2.1 mm) and gradient elution (MeCN/0.1% (v/v) HCOOH (H_2_O) 5/95 for 1 min, going up to 98/2 in 15 min, then 98/2 for another 3 min; flow rate 0.2 mL min^−1^).

The siderophore ferricrocin (used as a standard) was isolated as the ferri-form from *Aspergillus fumigatus* and was kindly provided by Prof. H. Haas (Innsbruck Medical University, Austria). Ferrichrome C (used as a standard) was isolated as the ferri-form from *Aspergillus ochraceous* and was purchased from EMC microcollections GmbH, Tübingen, Germany.

### Determination of extracellular siderophore activity

For determination of the siderophore activity in culture supernatants of *A*. *benhamiae* wild type, Δ*hapX* mutant and *hapX*^*C*^ reconstituted strain the chrome azurol S (CAS) liquid assay was used as described [32]. A volume of 100 ml of AMM without iron or supplemented with 0.03 mM ferrous sulfate was inoculated with 10^8^ microconidia and incubated for 3 d, 4 d and 5 d at 30°C and 200 rpm. The supernatant was collected by filtration through Miracloth and an aliquot of 100 μl culture supernatant was mixed with 100 μl CAS assay solution prepared according to Schwyn and Neilands [33]. As a reference AMM without iron was used. After incubation for 1 h at room temperature the absorbance at 630 nm was measured with a microtiter plate reader (Infinite^®^ 200 PRO, Tecan, Switzerland). The percentage of siderophore activity was calculated by substracting the sample absorbance values from the reference according to the formula [(A_r_-A_s_)/A_r_] x 100. The experiments were run in three biological replicates.

### Isolation of RNA and quantitative real-time reverse transcription-PCR (qRT-PCR)

For RNA isolation fungal mycelium was harvested after cultivation in AMM during iron-replete conditions (+Fe), iron starvation (-Fe, 0.03 mM FeSO_4_) and high iron conditions (hFe, 3 mM FeSO_4_) for 5 d at 30°C and 200 rpm. For short-term iron stress the mycelium was grown for 3 d at 30°C and 200 rpm and shifted from -Fe to +Fe for 1 h (sFe). The frozen mycelium was ground with mortar and pestle and subsequently the RNeasy Plant Mini Kit (QIAGEN, Venlo, Netherlands) was used for total RNA isolation according to the manufacturer’s instructions. The quality and quantity of RNA was determined with a NanoDrop 1000 Spectrophotometer (Thermo Fisher Scientific, Waltham, USA). For complete digestion of DNA 1000 ng RNA were treated with the TURBO DNA-*free*^™^ Kit (Ambion^®^, Thermo Fisher Scientific, Waltham, USA) and the purified RNA was used for first strand cDNA synthesis with oligo d(t)_23_ VN primer (New England Biolabs, Ipswich, USA) and RevertAid Reverse Transcriptase (Thermo Fisher Scientific, Waltham, USA) according to the manufacturer’s instructions. The qRT-PCR experiments were performed with the StepOnePlus Real-Time PCR System (Applied Biosystem, Thermo Fisher Scientific, Waltham, USA). Gene-specific primers (S2 Table) were designed with the software Primer3 [34]. The actin gene of *A*. *benhamiae* (ARB_04092) was chosen as internal control for normalization. Quantitative RT-PCR products were obtained using MyTaq HS Mix 2x (Bioline, London, UK) and EvaGreen (Biotium, Hayward, USA) as fluorescent dye. PCR conditions were 95°C for 2 min followed by 40 cycles with 15 s at 95°C, 20 s at 60°C, 15 s at 72°C and a final step at 95°C for additional 15 s. For each primer pair a standard curve with serial dilutions of genomic DNA of *A*. *benhamiae* in technical triplicates was determined and the primer efficiency (100% ±10) was used for the calculation of transcript levels by the method described by Pfaffl et al. [35]. Transcript levels are presented relative to those of *A*. *benhamiae* wild type during iron-replete conditions. The experiments were run in three biological replicates with technical duplicates.

### Growth on keratin substrates

Human hair and finger nails as well as keratin powder from hooves and horns (MP Biomedicals Germany GmbH, Eschwege, Germany) were used for the analysis of fungal growth on keratin substrates. Human scalp hair from a child and finger nails from a healthy female donor were cut from the donors themselves or the next of kin in their domestic home. The provided hair and clipped finger nail samples were autoclaved. Hair, nails and keratin powder were placed on water agar plates and inoculated with 3 plugs of fresh fungal mycelium from SAB agar plates. After incubation at 22°C for 25 d (keratin powder), 30 d (nails) and 40 d (hair) in the dark, mycelia formation on the keratin substrates was documented.

### Ethics Statement

The study did not include any diagnostic procedure or therapeutic method. Furthermore, the sample collection was non-invasive (the physical integrity of the donor was maintained) and did not intrude into the privacy of the donor. Based on the regulations of the ethics commission at the Jena University Hospital, Jena (Germany), an approval of this study was not necessary in this case. Only human material (scalp hair and clipped finger nails) from voluntary donors were used. Additionally, the donors or the next of kin have provided written consent for the use of the samples for research and publication. The study does not contain any patient data. Only the first author had access to any potentially identifying donor information. Identifying details are omitted in the manuscript.

## Results

### Identification of *A*. *benhamiae* HapX homologue

BLASTP search revealed a single HapX homologue in the genome of *A*. *benhamiae* which has significant similarity to HapX of *A*. *nidulans* (49% identity) and *A*. *fumigatus* (48% identity). *A*. *benhamiae hapX* is encoded by an open reading frame of 1425 bp with a deduced protein of 474 amino acids. Furthermore, HapX of *A*. *benhamiae* displays all the typical characteristics which are common to this class of transcription factors, including the basic region leucine zipper (bZIP) and coiled-coil domains mediating DNA-binding, an N-terminal CCAAT-binding complex (CBC) domain, which is essential for interaction with the CBC subunit HapE [18] and four conserved cysteine-rich regions (CRR) (S1 Fig). In *A*. *fumigatus*, two of the four CRR are known to be involved in detoxification of iron excess [22].

### Generation of *A*. *benhamiae* Δ*hapX* mutants and reconstituted strains

To assess the functional role of HapX, Δ*hapX* mutants were generated in the wild-type strain *A*. *benhamiae* LAU2354-2 by targeted *hapX* gene deletion with the *hph* resistance cassette (Fig 1A). To ensure that the observed phenotypes were a result of the deletion of *hapX*, the Δ*hapX* mutants were complemented with a copy of the wild-type *hapX* gene (Fig 1B). Southern blot analysis confirmed the site-directed insertion of the linear DNA cassettes (Fig 1C). The deletion strain AbenHAPXM1A (Δ*hapX*) and the reconstituted strain AbenHAPXK1A (*hapX*^*C*^) were used for further analysis.

### HapX is important for growth, conidiation and hyphal pigmentation during iron starvation

**Fig 2:**
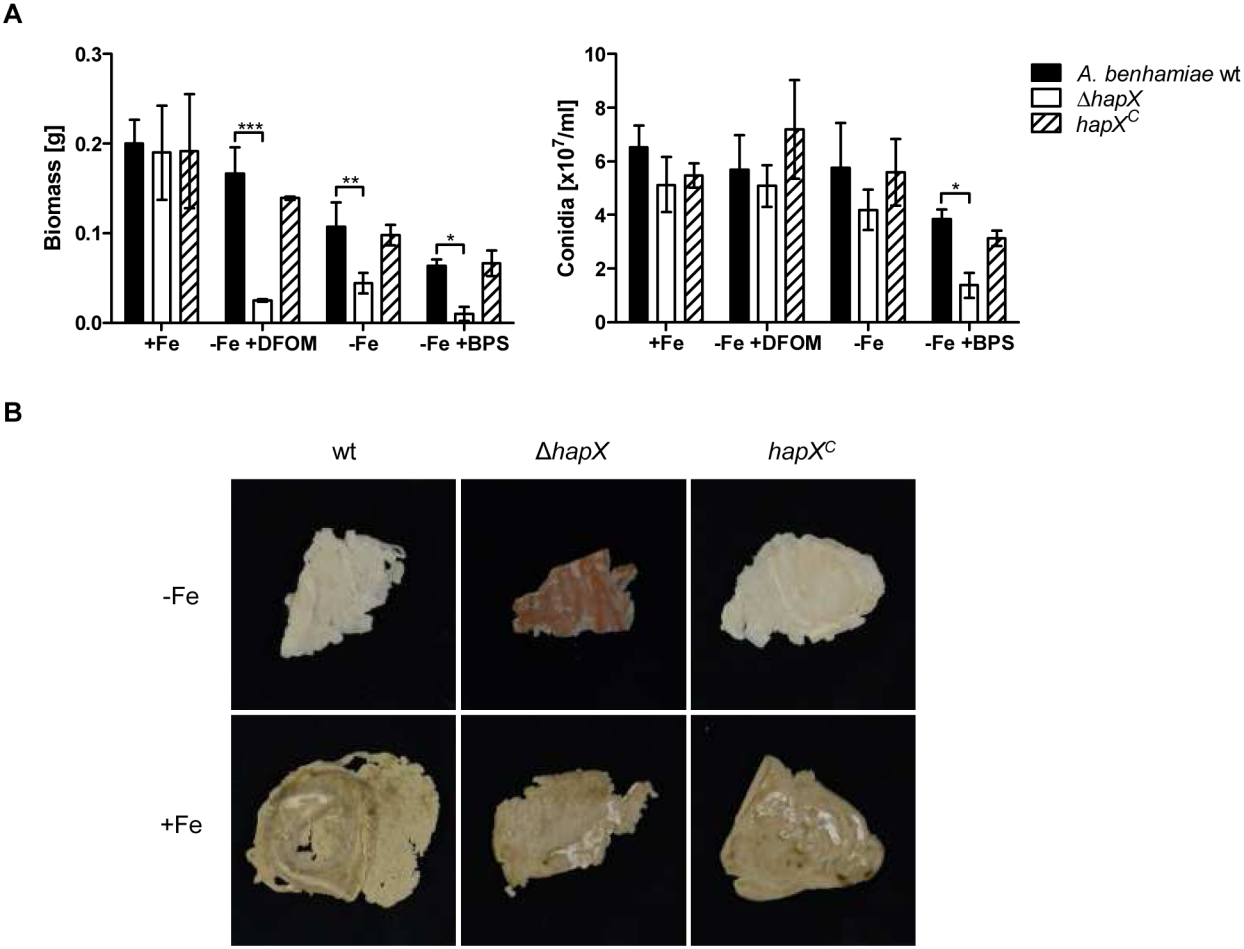
HapX of *A*. *benhamiae* is important for growth, conidiation and hyphal pigmentation during iron starvation. (A) Cultivation of *A*. *benhamiae* wild type, Δ*hapX* mutant and *hapX*^*C*^ reconstituted strain in AMM during iron-replete conditions (+Fe), iron limitation (-Fe), harsh iron starvation (-Fe +BPS) and in the presence of the xenosiderophore deferoxamine (-Fe + DFOM). Data represent the means and standard deviations of three biological replicates. The differences between wild type and Δ*hapX* mutant were statistically significant during iron starvation and in the presence of BPS and DFOM (2way ANOVA; * significant at P < 0.05, ** significant at P < 0.01, *** significant at P < 0.001). (B) Growth of the fungal strains in AMM during iron-replete conditions (+Fe) and iron starvation (-Fe) for 5 d at 30°C led to the formation of a reddish pigmented mycelium of Δ*hapX* mutant, particularly during iron deficiency.

Analysis of fungal growth and conidiation revealed reduced growth and decreased conidiation of the Δ*hapX* mutant during iron starvation, but not during iron-replete conditions (Fig 2). The biomass production of the Δ*hapX* mutant in liquid culture was significantly reduced during iron starvation and in the presence of the ferrous iron chelator BPS (Fig 2A). In contrast to *A*. *benhamiae* wild type and *hapX*^*C*^, growth of the Δ*hapX* mutant was highly impaired when the medium was supplemented with the xenosiderophore DFOM (Fig 2A). Additionally, loss of HapX led to a decrease of conidiation during iron starvation (-Fe) and in the presence of the iron chelator BPS but not during iron-replete conditions or in the presence of DFOM (Fig 2A). Furthermore, the mycelium of the Δ*hapX* mutant showed a reddish pigmentation during iron-depleted conditions probably due to the accumulation of iron-free precursors of heme (Fig 2B). By contrast, biomass and mycelial pigmentation of the Δ*hapX* mutant was comparable to the wild type during iron-replete conditions. Complementation of the *hapX* gene, resulting in the *hapX*^*C*^ reconstituted strain, restored the phenotype of *A*. *benhamiae* wild type in all experiments.

### HapX is necessary for the regulation of siderophore biosynthesis during iron starvation

**Fig 3:**
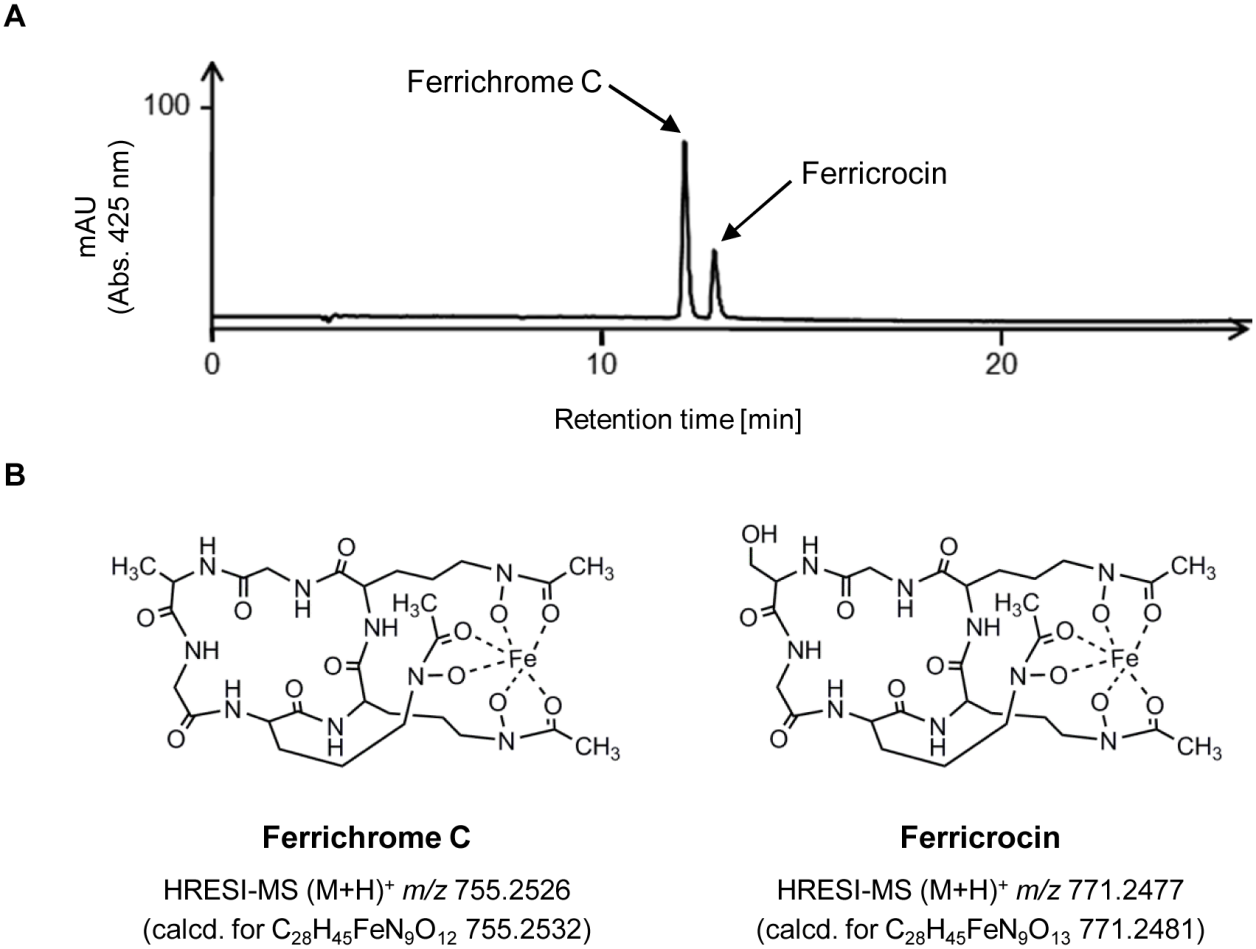
Identification of siderophores produced by *A*. *benhamiae*. (A) HPLC chromatogram of the lyophilized culture supernatants of *A*. *benhamiae*. Ferrichrome C and ferricrocin were identified as extracellular siderophores. (B) Chemical structures and molecular masses of the siderophores ferrichrome C and ferricrocin produced by *A*. *benhamiae*.

In order to identify the intra- and extracellular siderophores of *A*. *benhamiae* the supernatant and the mycelia of fungal cultures were separately extracted and analyzed by HPLC-PDA and HPLC-HRESIMS. Trace amounts of compounds with a molecular mass of *m/z* 753 (M-H^−^) and *m/z* 769 (M-H^−^) were found by MS analyses. MS/MS analyses and dereplication with commercial databases suggested a potential identity of the compounds with the hydroxamate-type siderophores ferrichrome C and ferricrocin, respectively. To unequivocally prove the structures, their UV spectra, HRESIMS-spectra, MS/MS fragmentation pattern as well as HPLC retention times were compared to authentic standards (S2–S6 Figs). As a result, ferrichrome C and ferricrocin could be identified as siderophores of *A*. *benhamiae* (Fig 3).

**Fig 4:**
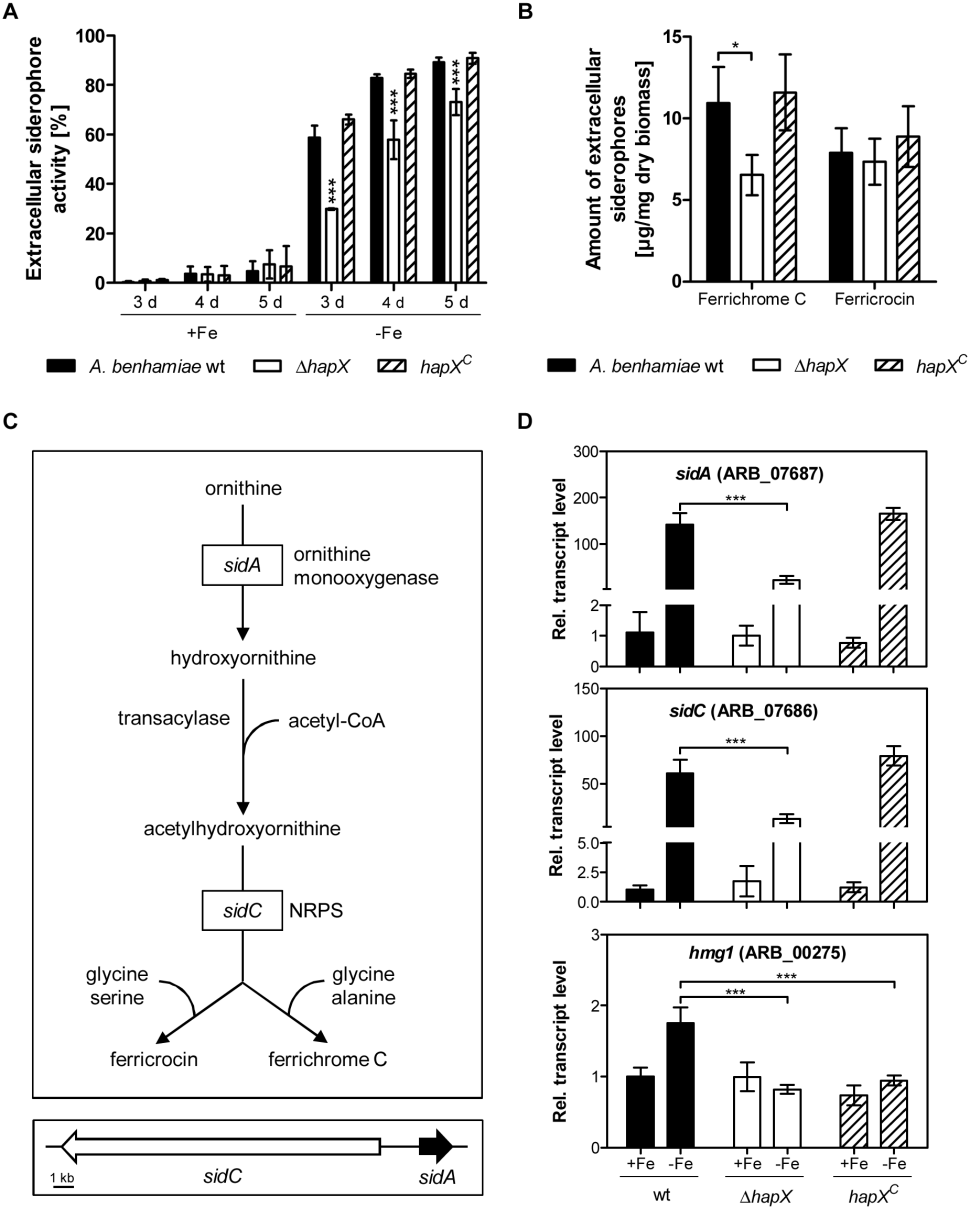
HapX-dependent siderophore biosynthesis of *A*. *benhamiae* during iron starvation. (A) Quantification of extracellular siderophores produced by *A*. *benhamiae* wild type, Δ*hapX* mutant and *hapX*^*C*^ reconstituted strain during iron starvation (-Fe) and iron sufficiency (+Fe). Data represent the means and standard deviations of three biological replicates. The differences between wild type and Δ*hapX* were statistically significant during iron starvation (2way ANOVA; *** significant at P < 0.001). (B) Quantification of the extracellular siderophores ferrichrome C and ferricrocin in supernatant extracts of *A*. *benhamiae* wild type, Δ*hapX* mutant and *hapX*^*C*^ reconstituted strain after cultivation for 5 d at 30°C during iron starvation by HPLC analysis. Data represent the means and standard deviations of three biological replicates. The differences between wild type and Δ*hapX* were statistically significant for ferrichrome C (2way ANOVA; * significant at P < 0.05) (C) Postulated biosynthesis pathway of the siderophores ferricrocin and ferrichrome C (based on the information from *A*. *fumigatus*) and genomic organization of the genes *sidC* (ARB_07686) and *sidA* (ARB_07687) of *A*. *benhamiae*. (D) Quantitative RT-PCR analysis of the transcript level of the genes *sidA* (ornithine monooxygenase), *sidC* (NRPS) and *hmg1* (HMG-CoA reductase) of *A*. *benhamiae* wild type, Δ*hapX* mutant and *hapX*^*C*^ reconstituted strain during iron starvation (-Fe) and iron-replete conditions (+Fe). Transcript levels are presented relative to those of *A*. *benhamiae* wild type during iron-replete conditions. Data represent the means and standard deviations of three biological replicates. The differences between wild type and Δ*hapX* were statistically significant during iron starvation (2way ANOVA; *** significant at P < 0.001).

The extracellular siderophores produced by *A*. *benhamiae* wild type, Δ*hapX* mutant and *hapX*^*C*^ reconstituted strains were quantified by using the CAS liquid assay (Fig 4A). During iron starvation, the extracellular siderophore production of the Δ*hapX* mutant was decreased in comparison to the wild type. However, all strains showed an increase of extracellular siderophore activity over time. In contrast, the siderophore production of *A*. *benhamiae* wild type, Δ*hapX* mutant and *hapX*^*C*^ reconstituted strain was almost abolished during iron-replete conditions.

Quantification of extracellular siderophores by HPLC analysis revealed a significantly reduced amount of secreted ferrichrome C in the Δ*hapX* mutant in comparison to the wild type (Fig 4B). By contrast, no difference between the wild type and Δ*hapX* mutant strain was observed for the extracellular concentration of ferricrocin (Fig 4B).

Homologues of proteins involved in the biosynthesis of the siderophores fusarinine C and TAFC were found in *A*. *benhamiae* by comparative genomic analysis with *A*. *fumigatus* (S3 Table). However, fusarinine C and TAFC were not identified in extracts of *A*. *benhamiae* culture supernatants or mycelium. Furthermore, homologous genes of the *A*. *fumigatus* TAFC esterase EstB, acetyltransferase SidG and siderophore iron transporter MirB are not clustered in *A*. *benhamiae* (S3 Table). Interestingly, the siderophore biosynthesis genes *sidC* (non-ribosomal peptide synthetase; ARB_07686) and *sidA* (ornithine monooxygenase; ARB_07687) of *A*. *benhamiae* are clustered (Fig 4C). Quantitative RT-PCR analysis of the genes *sidC* and *sidA* displayed that the transcript level of both genes is highly decreased in the Δ*hapX* mutant in comparison to the wild type during iron starvation (Fig 4D). In some fungi a link between siderophore biosynthesis and the isoprenoid biosynthesis pathway has been demonstrated [36, 37]. The 3-hydroxy-3-methyl-glutaryl-CoA (HMG-CoA) reductase is an important enzyme for isoprenoid biosynthesis. HMG-CoA reductase is encoded by the gene *hmg1* and its expression is dependent on the iron availability, but only in fungi which produce mevalonate-derived siderophores such as fusarinine C or TAFC [36]. Quantitative RT-PCR analysis of the gene *hmg1* showed that the transcript level of *hmg1* is slightly upregulated in *A*. *benhamiae* wild type during iron starvation. By contrast, no differences in the transcript level of *hmg1* were observed during iron-replete conditions and iron starvation in the Δ*hapX* mutant and *hapX*^*C*^ reconstituted strains (Fig 4D).

### HapX is required for transcriptional repression of genes involved in iron-dependent pathways during iron starvation

**Fig 5:**
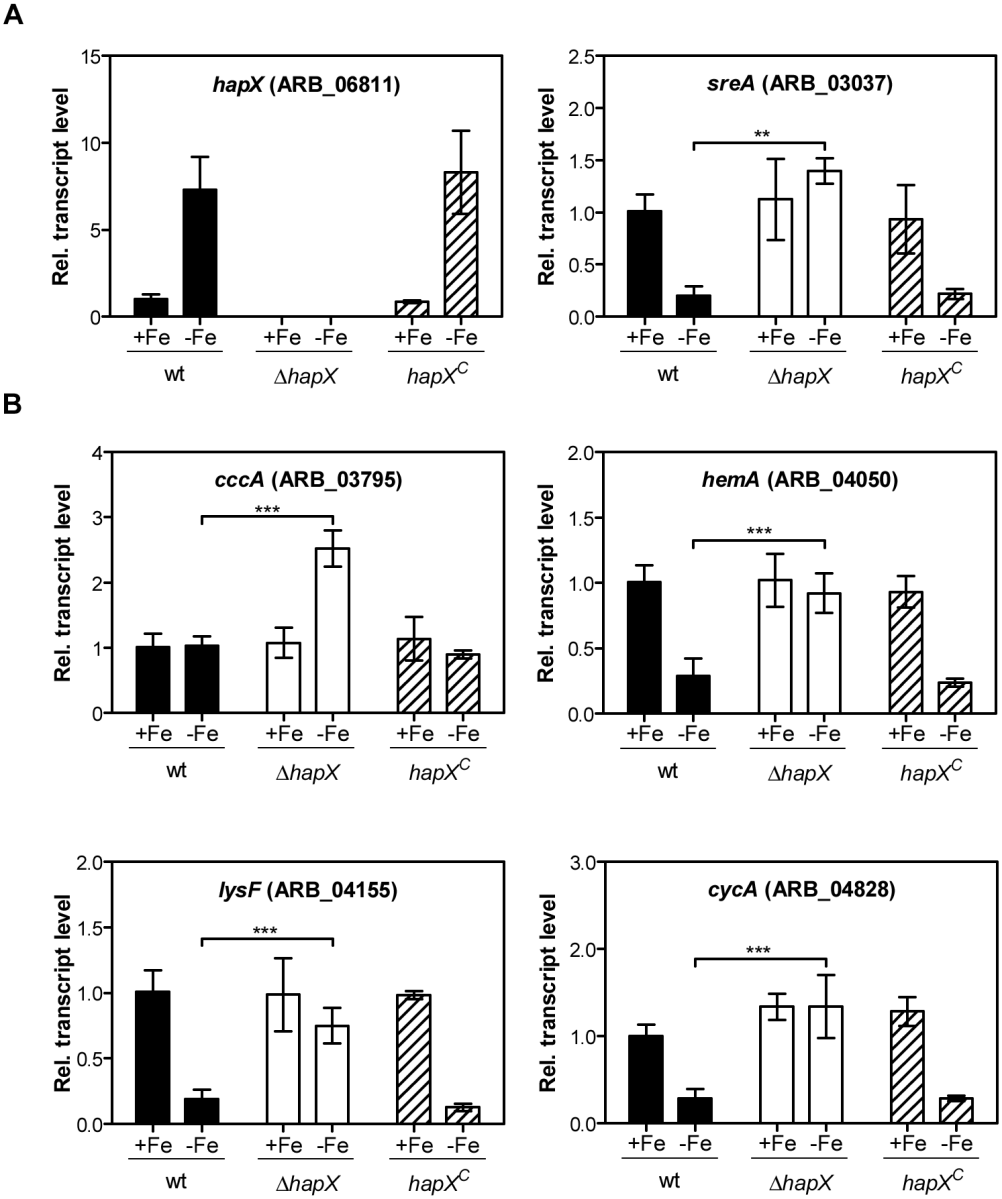
HapX of *A*. *benhamiae* is important for transcription of iron regulatory genes during iron limitation. (A) Transcript levels of transcription factors HapX and SreA during iron starvation (-Fe) and iron-replete conditions (+Fe). (B) Transcript levels of the genes *cccA*, *hemA*, *lysF* and *cycA* during iron starvation (-Fe) and iron-replete conditions (+Fe). Transcript levels measured by quantitative RT-PCR analysis are presented relative to those of *A*. *benhamiae* wild type during iron-replete conditions. Data represent the means and standard deviations of three biological replicates. The differences between wild type and Δ*hapX* mutant were statistically significant during iron starvation (2way ANOVA; ** significant at P < 0.01, *** significant at P < 0.001).

Next, we investigated the role of the transcriptional regulators HapX and SreA of *A*. *benhamiae* in the presence or absence of iron by qRT-PCR. During iron starvation, the transcript level of *hapX* was highly upregulated in *A*. *benhamiae* wild type in comparison to iron-replete conditions, which indicates that *hapX* transcription is repressed by iron (Fig 5A). By contrast, the transcript level of *sreA* was downregulated in *A*. *benhamiae* wild type, but significantly increased in the Δ*hapX* mutant during iron starvation compared to iron-replete conditions which shows that HapX represses the *sreA* gene during iron starvation (Fig 5A).

Representative genes from known iron consuming pathways were chosen for further transcriptional analysis of HapX during iron starvation and iron-replete conditions. In *A*. *fumigatus* the *cccA* gene encodes a vacuolar iron importer which mediates the import of iron into the vacuole to avoid toxic levels of this metal in the cytosol [25]. The genes *hemA* (5-aminolevulinic acid synthase), *cycA* (cytochrome C) and *lysF* (homoaconitase) are involved in heme biosynthesis, respiration and lysine biosynthesis, respectively [18, 19]. The transcript levels of *cccA*, *hemA*, *cycA* and *lysF* were significantly increased in the Δ*hapX* mutant during iron starvation, but not during iron-replete conditions in comparison to the wild type (Fig 5B).

### HapX is involved in iron detoxification

**Fig 6:**
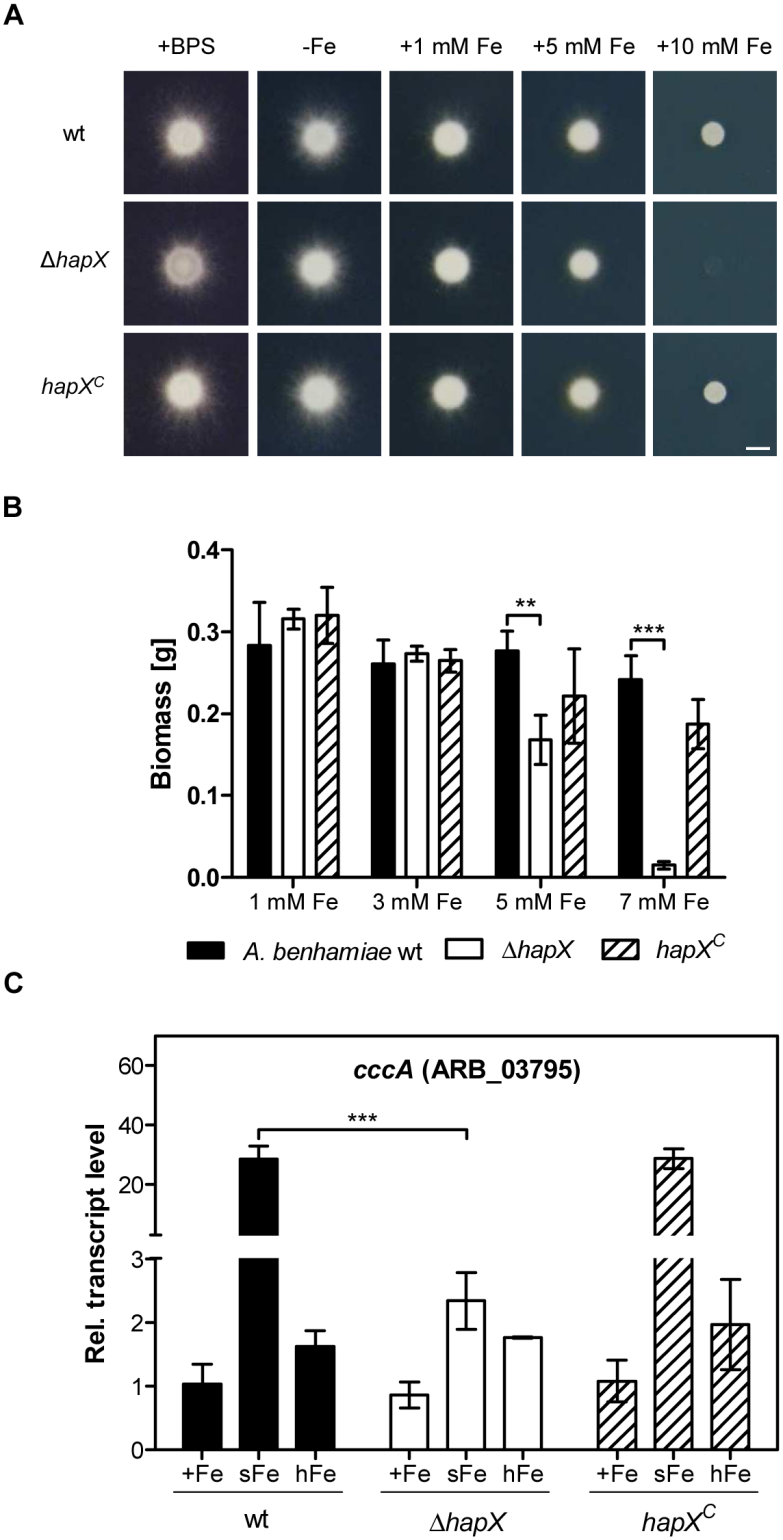
HapX of *A*. *benhamiae* under high iron concentrations. (A) Cultivation of *A*. *benhamiae* wild type, Δ*hapX* mutant and *hapX*^*C*^ reconstituted strain during iron starvation (-Fe), high iron concentrations (1–10 mM FeSO_4_) and in the presence of the iron chelator BPS on solid medium for 7 d at 30°C. Scale bar represents 5 mm. (B) Cultivation of *A*. *benhamiae* wild type, Δ*hapX* mutant and *hapX*^*C*^ reconstituted strain in AMM with high iron concentrations (1–7 mM FeSO_4_). Data represent the means and standard deviations of three biological replicates. The differences between wild type and Δ*hapX* mutant were statistically significant between 5 mM and 7 mM Fe (2way ANOVA; ** significant at P < 0.01, *** significant at P < 0.001). (C) Quantitative RT-PCR analysis of the *cccA* gene under different iron concentrations. The mycelium was cultivated under high iron concentrations (hFe) or shifted for 1 h from -Fe to +Fe (sFe). Data represent the means and standard deviations of three biological replicates. The differences between wild type and Δ*hapX* mutant were statistically significant during sFe (2way ANOVA; *** significant at P < 0.001).

In addition to the characterization of HapX during iron starvation, the role of this transcription factor in the presence of iron excess was analyzed. Cultivation of *A*. *benhamiae* wild type, Δ*hapX* mutant and *hapX*^*C*^ reconstituted strains on agar plates and in liquid medium resulted in a strong growth defect of the Δ*hapX* mutant in the presence of 5 to 10 mM FeSO_4_ (Fig 6A and 6B). Quantitative RT-PCR analysis of the vacuolar iron importer encoding gene *cccA* showed that the transcript level of *cccA* was highly upregulated during a shift from iron starvation to 0.03 mM FeSO_4_ for 1 h (sFe) in *A*. *benhamiae* wild type but not in the Δ*hapX* mutant (Fig 6C). By contrast, no significant differences in the transcript level of *cccA* in *A*. *benhamiae* wild type and the Δ*hapX* mutant were observed during growth in medium supplemented with 3 mM FeSO_4_ (hFe) (Fig 6C).

### HapX is dispensable for growth on keratin substrates

**Fig 7:**
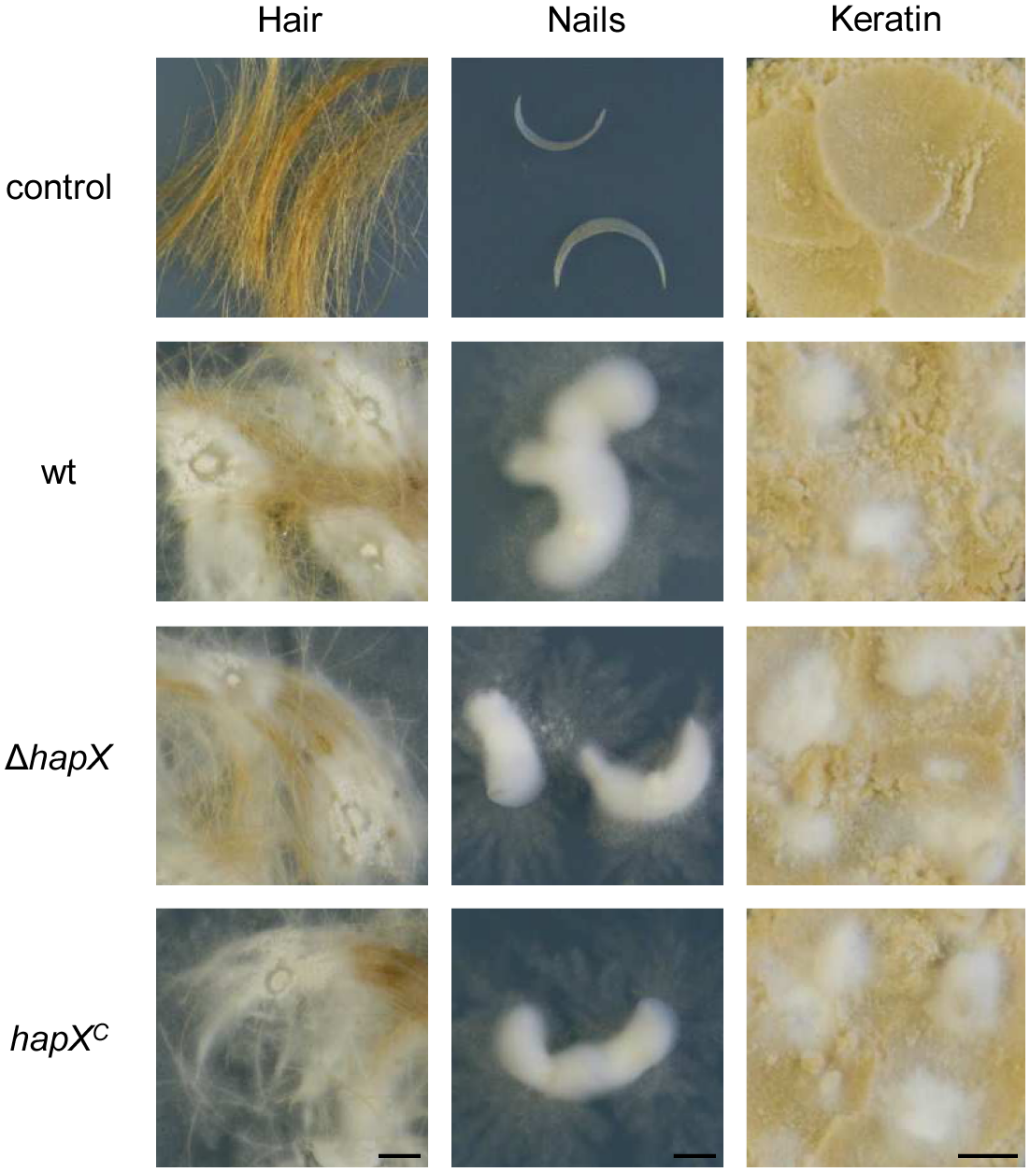
HapX of *A*. *benhamiae* is dispensable for growth on keratin substrates. Cultivation of *A*. *benhamiae* wild type, Δ*hapX* mutant and *hapX*^*C*^ reconstituted strain on human hair, finger nails and keratin powder derived from hooves and horns. Scale bar represents 5 mm.

To date, only few models are available for testing putative virulence factors of dermatophytes [38]. Besides the application of animal models in guinea pig and mouse [39, 40], keratinized host tissues, including hair and nails, have been used for the analysis of putative pathogenicity associated factors in dermatophytes [12, 41]. To test whether the transcription factor HapX plays a role during infection, *in vitro* growth of *A*. *benhamiae* wild type, Δ*hapX* mutant and *hapX*^*C*^ reconstituted strain on human hair and nails as well as on keratin powder derived from hooves and horns was analyzed. No growth differences were observed between *A*. *benhamiae* wild type, Δ*hapX* mutant and *hapX*^*C*^ reconstituted strain during infection of all tested keratin substrates (Fig 7).

## Discussion

To overcome iron deficiency, many pathogenic fungi produce, release and take up siderophores. The chemical structure of siderophores enable them to chelate ferric iron and even extract iron from transferrin, lactoferrin or ferritin [42, 43]. The production of siderophores is usually transcriptionally regulated depending on iron availability.

As shown here, the emerging human pathogenic dermatophyte *A*. *benhamiae* produces the hydroxamate siderophores ferricrocin and ferrichrome C as intra- and extracellular siderophores. Both siderophores were isolated from the culture supernatant of the dermatophytes *Microsporum gypseum*, *Microsporum audouinii*, *M*. *canis* as well as *T*. *rubrum* [44]. Usually, filamentous fungi use ferrichrome-type siderophores for intracellular iron distribution and iron storage [45] as shown for *A*. *nidulans*, *A*. *fumigatus*, *Fusarium graminearum*, *F*. *oxysporum*, *Neurospora crassa* and *Magnaporthe grisea* [29, 46–50]. For iron acquisition, however, most fungi produce and secrete different hydroxamate siderophores, such as fusarinines and coprogens. The extracellular siderophores fusarinine C and triacetylfusarinine C (TAFC), for instance, are produced by *A*. *nidulans* and *A*. *fumigatus* [46, 51, 52], exclusively fusarinine C by *Fusarium roseum* and *F*. *oxysporum* [29, 53] and coprogens by *N*. *crassa* and *M*. *grisea* [49, 50]. In contrast to other fungi, dermatophytes appear to produce ferrichromes for both intra- and extracellular use. In this context, it is interesting to note that secretion of ferrichrome-type siderophores has been described for the yeast *Schizosaccharomyces pombe* [54], the basidiomycete *Ustilago maydis* [55] and the filamentous fungi *F*. *roseum* and *F*. *oxysporum* [29, 53]. In the presence of the bacterial siderophore deferoxamine biomass production of the *A*. *benhamiae* wild-type strain was increased under iron limitation compared to iron-depleted conditions which indicates that *A*. *behamiae* is able to use xenosiderophores as iron source. Similar results were reported for species of *Paracoccidioides* [56]. By contrast, growth of the Δ*hapX* mutant of *A*. *benhamiae* was highly negatively affected by the presence of deferoxamine. This result suggests that the Δ*hapX* mutant was unable to use xenosiderophore for iron acquisition, which may be caused by a defect in the siderophore uptake system in this mutant. Uptake of the ferri-form of the siderophores is mediated by siderophore-iron transporters (SITs). Although SITs are quite conserved in siderophore producing and non-producing fungal species only a few SITs have been identified and functionally characterized, so far (reviewed in [24]). In dermatophytes, SITs responsible for uptake of ferrichrome C and ferricrocin have not been identified yet.

The structure of ferricrocin differs from ferrichrome C only by a serine for alanine substitution. It has been suggested that both siderophores ferricrocin and ferrichrome C are synthesized by the ferrichrome-type NRPS SidC in *F*. *oxysporum* [29]. The genes encoding NRPS SidC and ornithine monooxygenase SidA of *A*. *benhamiae* are clustered which is typical for genes encoding components of common pathways in filamentous fungi. Similar to *U*. *maydis*, *S*. *pombe*, *F*. *graminearum* and *F*. *oxysporum* the genes *sidA* and *sidC* of *A*. *benhamiae* are probably bidirectionally transcribed from a common promoter region [29, 54, 57, 58]. In contrast, *sidA* and *sidC* of *A*. *nidulans* and *A*. *fumigatus* are located on different chromosomes [47, 59]. Conversely, the genes encoding TAFC esterase EstB, acetyltransferase SidG, siderophore iron transporter MirB and an ABC-transporter are clustered in *A*. *fumigatus* [60], but not in *A*. *benhamiae* (S3 Table). Although *Arthroderma* and *Aspergillus* both belong to the subclass Eurotiomycetidae, the organisation of their siderophore biosynthesis genes is obviously more similar to phylogenetically more distantly related phyla.

Deletion of *hapX* of *A*. *benhamiae* resulted in a lacking activation of the siderophore biosynthesis genes *sidA* and *sidC* during iron starvation and in a decreased production of extracellular ferrichrome C, but not ferricrocin. Similarly, the Δ*hapX* mutant of *A*. *fumigatus* displayed a downregulation of genes involved in siderophore biosynthesis and a decreased production of the siderophores ferricrocin and TAFC, but not fusarinine C during iron-limiting conditions [19]. Divergent from this observation, lack of HapX caused a reduced TAFC production but an increased level of ferricrocin and the *sidC* transcript during iron-depleted conditions in *A*. *nidulans* [18]. By contrast, inactivation of *hapX* in *F*. *oxysporum* led to elevated transcript levels of several siderophore genes and to an increased amount of intracellular, but not extracellular siderophores during iron starvation [29].

Transcriptional regulation of iron homeostasis and regulation of the siderophore system in *A*. *nidulans*, *A*. *fumigatus* and *S*. *pombe* is achieved by the transcription factors HapX and SreA (referred to as Php4 and Fep1 in *S*. *pombe*) which are interconnected by a negative feed-back loop [18, 19, 61, 62]. The data from our study indicate that the same model can be applied to *A*. *benhamiae*. SreA represses the expression of *hapX* and the siderophore system during iron sufficient conditions by an iron-sensing mechanism, while HapX represses *sreA* and activates the siderophore system during iron-limiting conditions resulting in efficient iron uptake and inhibition of iron-consuming pathways. As described above, deletion of *hapX* in *A*. *benhamiae*, *A*. *fumigatus* and *A*. *nidulans* resulted to some extent in a decreased siderophore production [18, 19]. At the same time, this result implies the existence of alternative mechanisms regulating the siderophore system. Besides HapX and SreA, the sterol regulatory element binding protein (SREBP) SrbA was shown to contribute to the activation of siderophore production in *A*. *fumgatus* [63]. Furthermore, the pH signaling transcription factor PacC, the gluconeogenesis-regulating transcription factors AcuK and AcuM and the mitogen-activated protein kinase (MAPK) MpkA have been suggested to be involved as well [64–67].

The main precursor for fungal siderophores is the non proteinogenic amino acid ornithine [45]. Additionally, the biosynthesis of fusarinine-type siderophores is linked to the isoprenoid biosynthesis pathway in *A*. *fumigatus*. The intermediate mevalonate produced by the HMG-CoA reductase serves as precursor for the biosynthesis of fusarinine C and TAFC [36]. In *A*. *fumigatus*, the transcript level of the gene encoding the HMG-CoA reductase, *hmg1*, is highly increased during iron starvation [36]. In contrast, the availability of iron did not significantly influence the transcript level of *hmg1* in *A*. *benhamiae*. This is in line with data from the non-siderophore producing fungi *S*. *cerevisiae*, *C*. *neoformans* and *C*. *albicans* [68–70]. Similarly, the presence or absence of iron did not affect the expression of *hmg1* in *U*. *maydis* [71] despite the fact that ferrichrome A biosynthesis is also linked to the isoprenoid biosynthesis pathway in *U*. *maydis* [37].

Besides altered regulation of siderophore biosynthesis, deletion of HapX in *A*. *benhamiae* resulted in decreased growth, conidiation and complete deregulation of genes from iron-dependent pathways such as vacuolar iron storage, amino acid metabolism, respiration and heme biosynthesis during iron limitation. The transcript level of *cccA* (vacuolar iron importer), *lysF* (homoaconitase), *cycA* (cytochrome C) and *hemA* (5-amino-levulinic acid synthase) was highly upregulated in the Δ*hapX* mutant during iron starvation. Similar results have been obtained for loss of HapX homologues in *A*. *nidulans*, *A*. *fumigatus*, *F*. *oxysporum*, *C*. *neoformans*, *C*. *albicans* and *S*. *pombe* [18, 19, 27–29, 61, 62]. Due to the activated heme biosynthesis during iron starvation, predictably the iron-free heme precursor protoporphyrin IX accumulated in the Δ*hapX* mutant of *A*. *benhamiae* causing a reddish pigmentation of the mycelium. The same has been previously reported from *A*. *nidulans*, *A*. *fumigatus* and *F*. *oxysporum* [18, 19, 29].

The transcription factor HapX is also essential for iron detoxification by activating the vacuolar iron importer CccA during high iron conditions [21, 25]. Consequently, deletion of *hapX* inhibited growth of *A*. *fumigatus*, *A*. *nidulans*, *F*. *oxysporum* and *A*. *benhamiae* in the presence of excess iron (this study, [22]). Similarly, the transcript level of *cccA* was highly upregulated during a shift from iron starvation to iron-replete conditions for one hour in *A*. *benhamiae* wild type, but not in the Δ*hapX* mutant. Interestingly, long-term periods of iron excess did not affect the transcript level of *cccA* in *A*. *benhamiae* which underlines the importance of the vacuolar iron importer during acute high iron stress. Distinct protein domains of HapX allow this Janus-type transcription factor to function as activator or repressor and consequently, to mediate both adaptation to iron starvation and iron resistance [22]. Additionally, it was found that the CBC-HapX complex of *A*. *nidulans* cooperatively binds to a bipartite DNA motif within the promoter of genes which are downregulated during iron limitation [23]. Characterization of this DNA motif in promoters of the genes *cycA*, *sreA*, *acoA* and *lysF* in *A*. *nidulans* resulted in the identification of the minimal motif 5’-GAT-3’, which is located at a distance of 11–12 bp downstream of the respective CCAAT box [23]. A similar motif is also evolutionary conserved in the *cccA* promoter of 28 fungi including species of *Aspergillus* and dermatophytes, *e*.*g*. *A*. *benhamiae* [22].

The critical role of iron acquistion in host-pathogen interactions has been demonstrated in various animal and plant pathogenic fungi. Siderophore-mediated iron uptake was shown to be essential for virulence of *A*. *fumigatus*, *C*. *albicans*, *F*. *oxysporum* and to a lesser extent in *C*. *neoformans* in murine infection models [19, 27–29, 47, 52, 72, 73]. Surprisingly, the Δ*hapX* mutant of *A*. *benhamiae* did not show a virulence defect during *in vitro* infection of hair and nails. A major difference between the human pathogenic fungi *A*. *fumigatus*, *C*. *albicans*, *C*. *neoformans* and *A*. *benhamiae* is the infectious life cycle. In contrast to *A*. *fumigatus*, *C*. *albicans* and *C*. *neoformans*, which are able to grow invasively in immunocompromised individuals, *A*. *benhamiae* is restricted to superficial growth on keratinized host structures such as skin (stratum corneum), hair and nails of humans and animals. We hypothesized that skin, hair and nails constitute a highly iron-restricted environment, but the ability of the Δ*hapX* mutant of *A*. *benhamiae* to grow on these keratinized structures might result from sufficient iron acquisition. In support of this idea, it has been described that iron is excreted by skin through desquamation of iron-loaded epithelial cells [74]. Furthermore, previous studies have shown that human epidermis, hair of children and finger nails contain variable amounts of iron [75–77]. It is possible that iron of these keratin substrates is easily accessable for dermatophytes by siderophores or alternative iron uptake mechanisms, which might explain the missing growth defect of the Δ*hapX* mutant of *A*. *benhamiae* on keratin. Besides siderophores, the *A*. *benhamiae* genome encodes proteins which represent homologues of iron permeases and oxidoreductases known to be involved in reductive iron assimilation (RIA) (S3 Table). These proteins may contribute to iron acquisition, too. Alternative mechanisms for iron acquisition such as low-affinity iron uptake systems have been described in *S*. *cerevisiae*, *C*. *neoformans* and *A*. *nidulans* [59, 78, 79]. Prerequisite for iron uptake by low-affinity mechanisms is the availability of ferrous iron which is the prevalent form of iron during acidic conditions. Human skin, scalp, hair shafts and nail plate have an acidic pH of 5.5 and below [80–82] which suggests that ferrous iron uptake mechanisms might play a role during superficial growth of *A*. *benhamiae*. Additionally, genes involved in iron homeostasis were not differentially regulated in the transcriptome of *A*. *benhamiae* during infection of human keratinocytes [13]. This result further supports the idea that siderophore-mediated iron uptake plays a minor role during dermatophyte infection.

## Conclusions

This study underlines the highly conserved role of the fungal-specific transcription factor HapX for adaptation to iron starvation and especially, its relevance for the downregulation of iron-consuming pathways and the activation of siderophore biosynthesis during iron deficiency in ascomycetous fungi. Furthermore, HapX is a virulence factor in many plant and human pathogenic fungi *in vivo*, but is redundant in *A*. *benhamiae* during *in vitro* infection of keratinized host tissues, which might reflect the different iron supply or requirements of fungi during their respective infectious life cycle.

## Supporting Information

S1 Fig Multiple sequence alignment of the HapX protein of *Aspergillus fumigatus* (AFUA_5G03920), *Arthroderma benhamiae* (ARB_06811) and *Trichophyton rubrum* (TERG_07733.3) using Clustal Omega (http://www.ebi.ac.uk/Tools/msa/clustalo/). The N-terminal CBC binding domain is indicated by bold letters, the bZIP domain is shaded in black, the coiled-coil domain is highlighted in grey and the four conserved cysteine-rich regions are indicated by black lines.
(TIF)

S2 Fig Extracted mass traces (ESI^+^). (A) Ferricrocin standard *m/z* 771. (B) Ferrichrome C standard *m/z* 755. (C) Mycelial extract of *A*. *benhamiae* wild type *m/z* 771. (D) Mycelial extract of *A*. *benhamiae* wild type *m/z* 755.
(TIF)

S3 Fig MS/MS spectrum of ferrichrome C reference. (TIF)

S4 Fig MS/MS spectrum of ferricrocin reference. (TIF)

S5 Fig MS/MS spectrum of ferrichrome C detected in the mycelial extract of *A*. *benhamiae*. (TIF)

S6 Fig MS/MS spectrum of ferricrocin detected in the mycelial extract of *A*. *benhamiae*. (TIF)

S1 Table Primers used for the generation of plasmids. (PDF)

S2 Table Primers used for qRT-PCR. (PDF)

S3 Table Putative homologues of proteins involved in iron homeostasis of *A*. *benhamiae*. (PDF)
