# Supplementary figures and images for: HapX Mediates Iron Homeostasis in the Pathogenic Dermatophyte Arthroderma benhamiae but Is Dispensable for Virulence
Source: PLoS One. 2016 Mar 9;11(3):e0150701. doi: 10.1371/journal.pone.0150701 (PMC4784894; doi:10.1371/journal.pone.0150701)

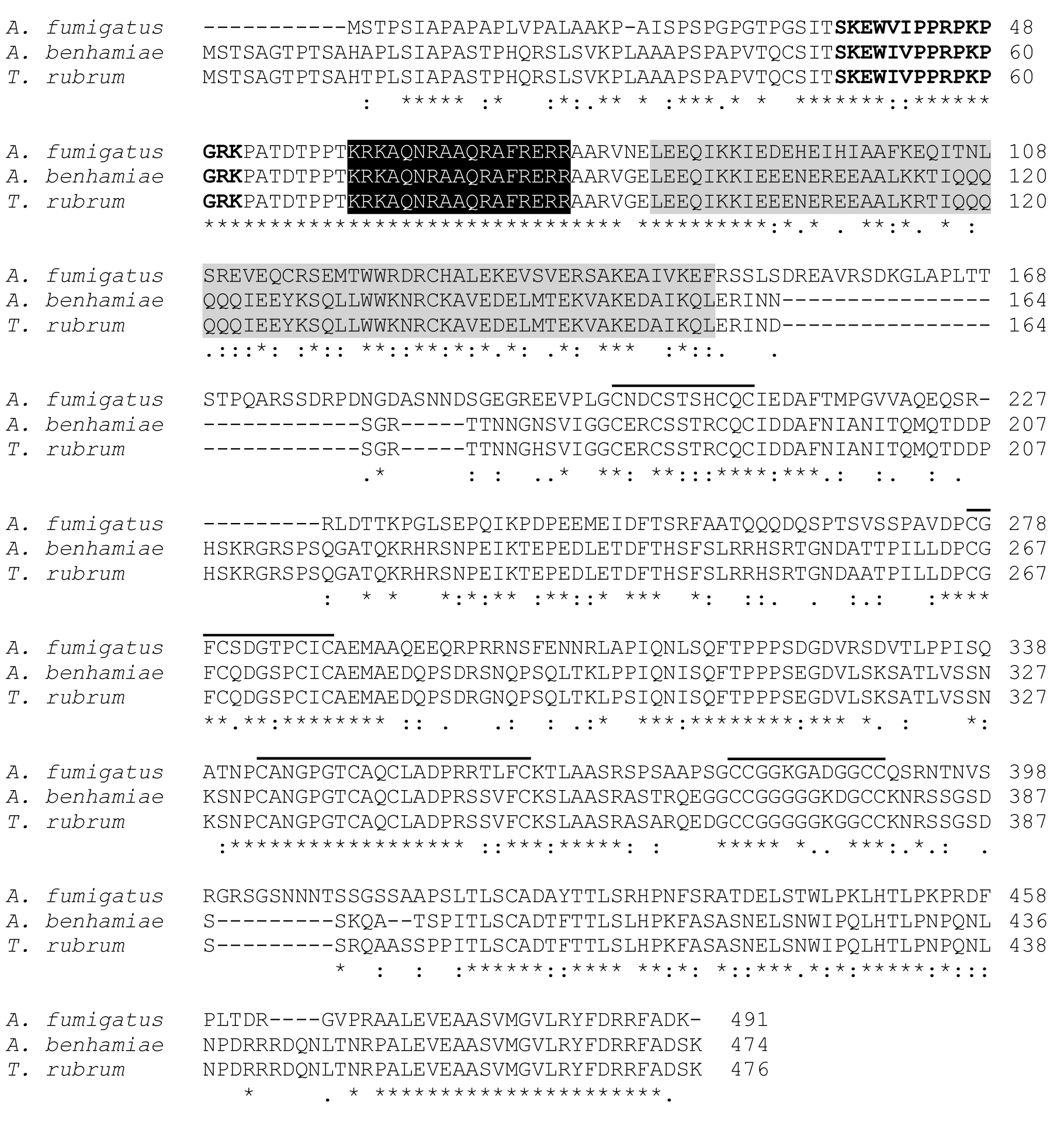

Supplement: S1 Fig — The N-terminal CBC binding domain is indicated by bold letters, the bZIP domain is shaded in black, the coiled-coil domain is highlighted in grey and the four conserved cysteine-rich regions are indicated by black lines. (TIF) [file pone.0150701.s001.tif]

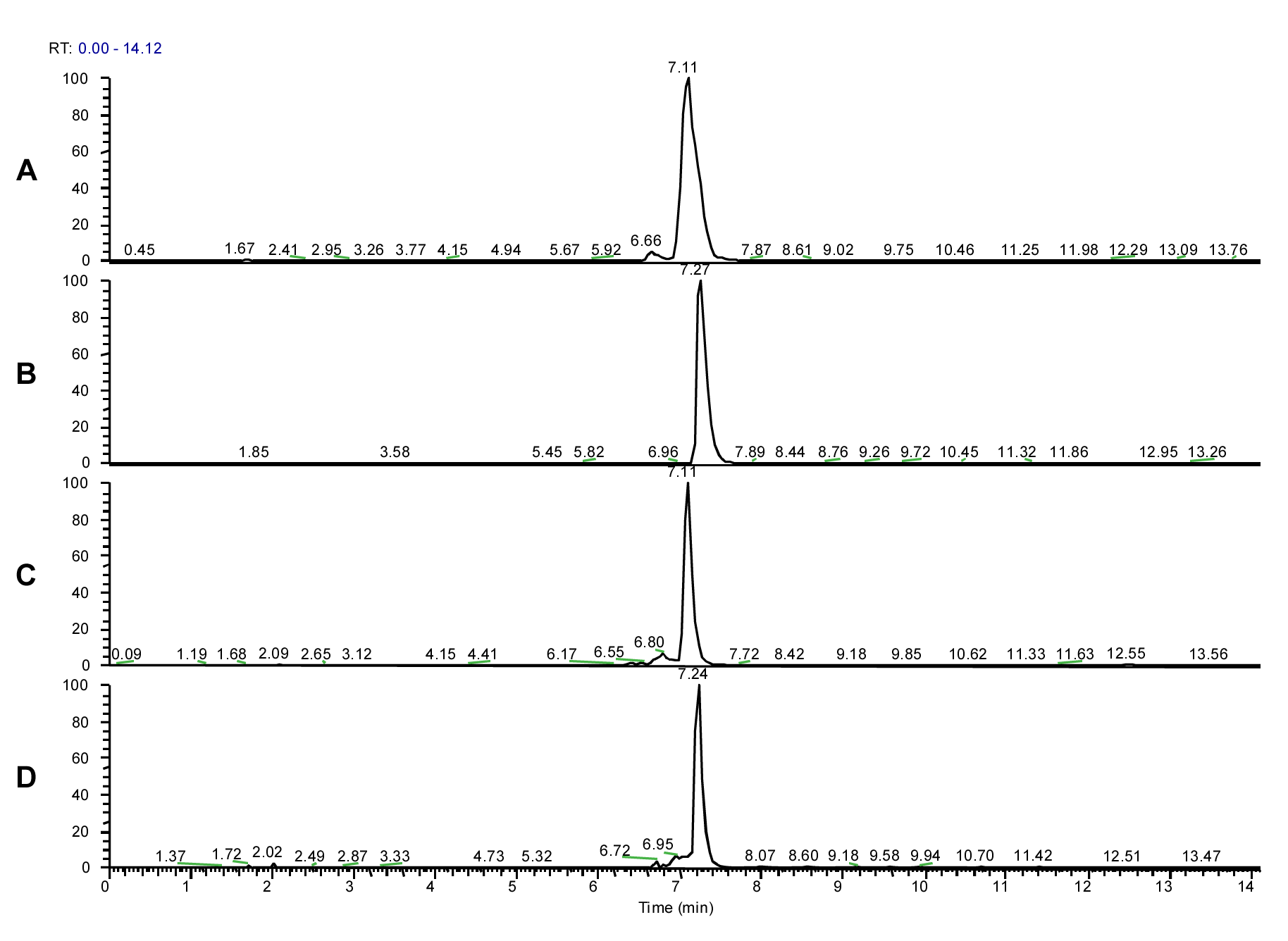

Supplement: S2 Fig — (A) Ferricrocin standard m/z 771. (B) Ferrichrome C standard m/z 755. (C) Mycelial extract of A. benhamiae wild type m/z 771. (D) Mycelial extract of A. benhamiae wild type m/z 755. (TIF) [file pone.0150701.s002.tif]

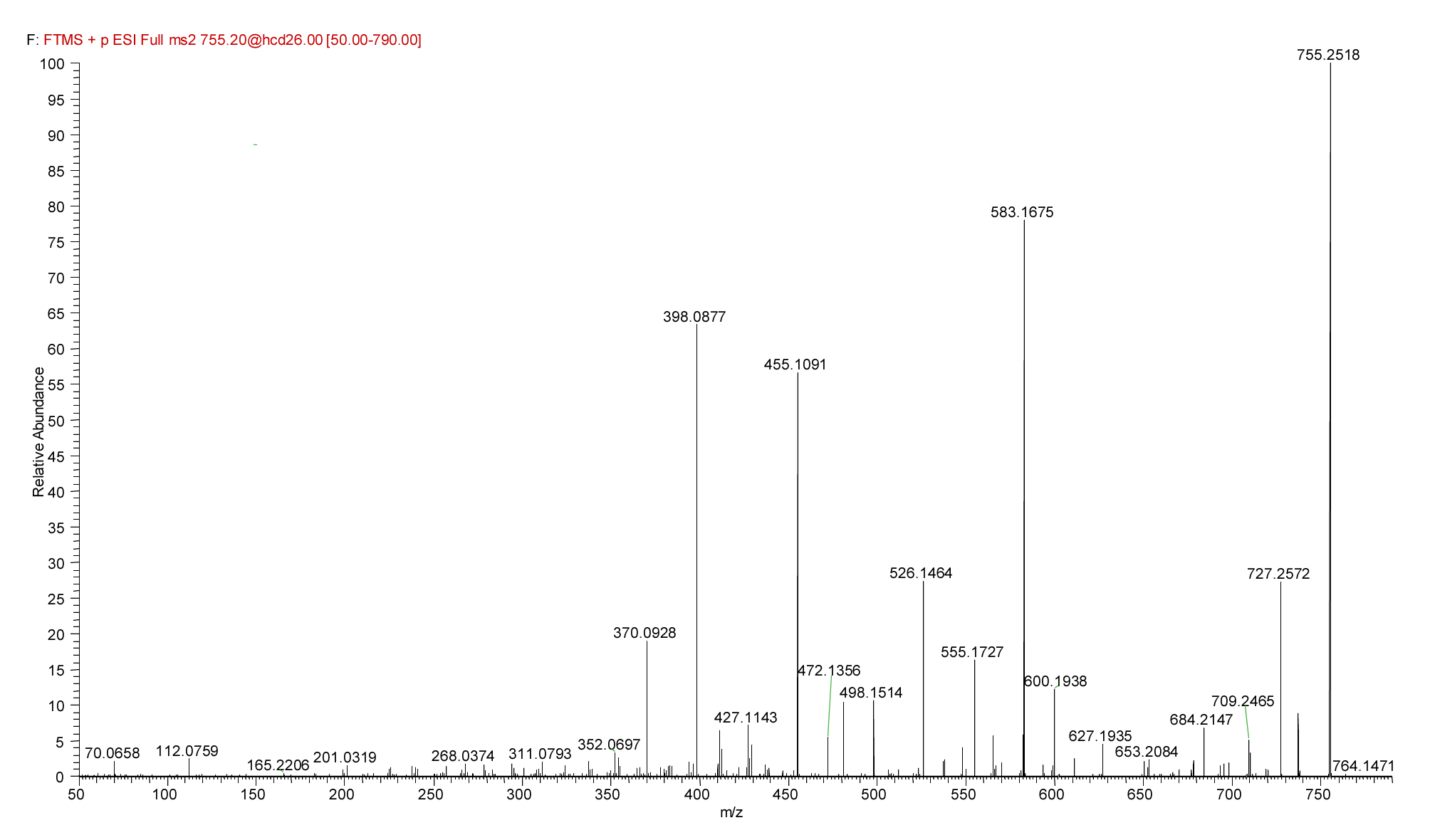

Supplement: S3 Fig — (TIF) [file pone.0150701.s003.tif]

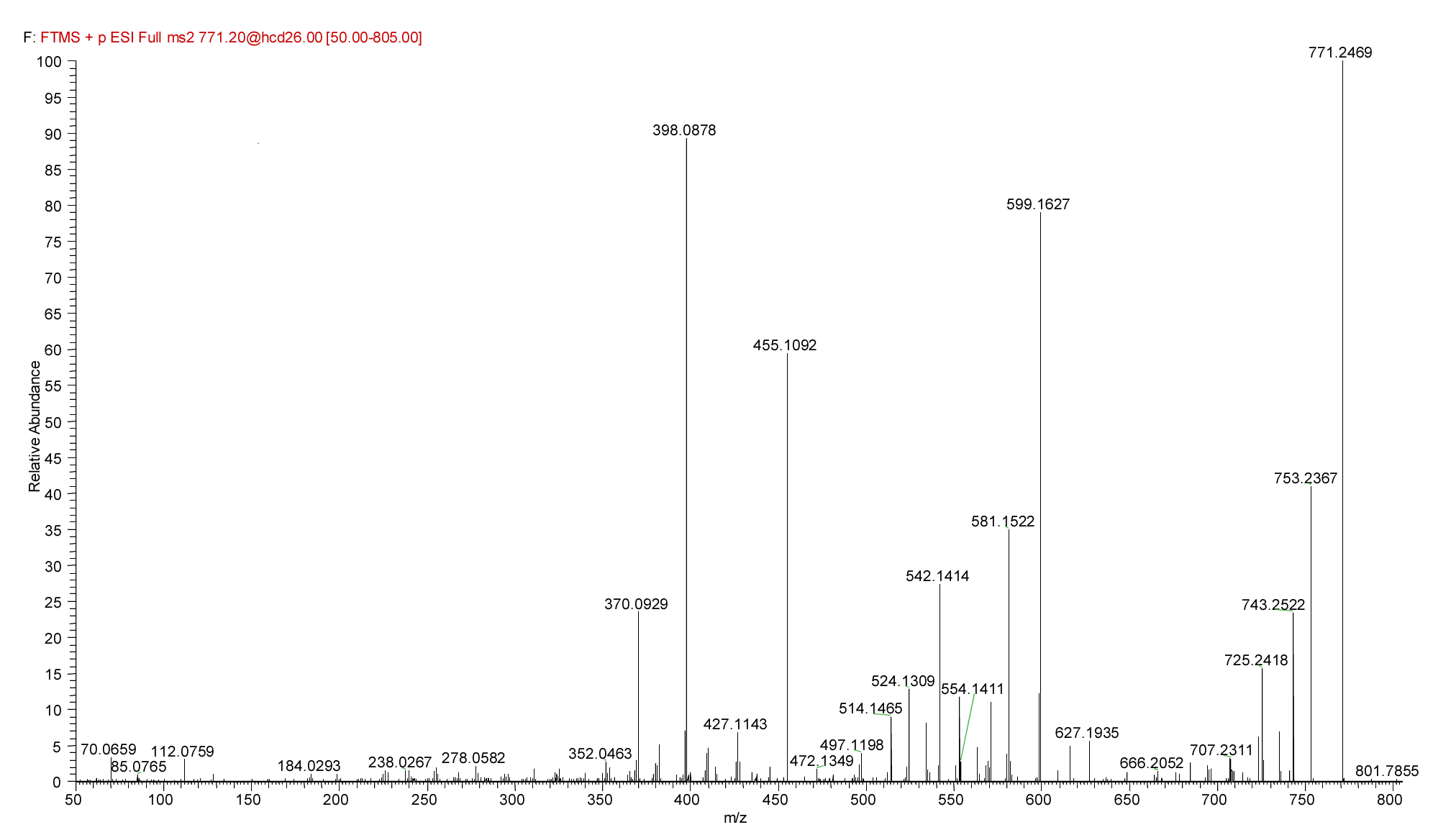

Supplement: S4 Fig — (TIF) [file pone.0150701.s004.tif]

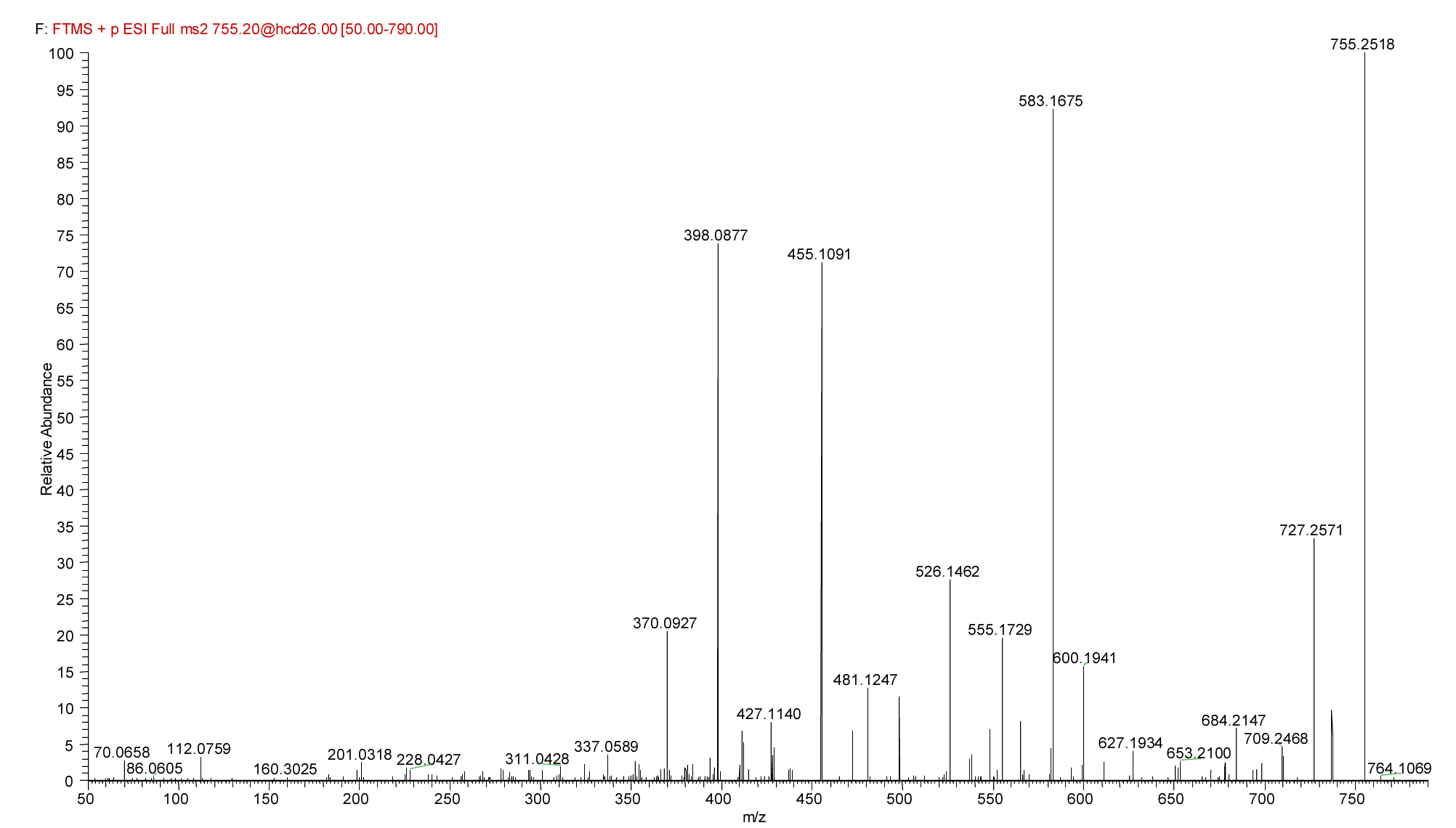

Supplement: S5 Fig — (TIF) [file pone.0150701.s005.tif]

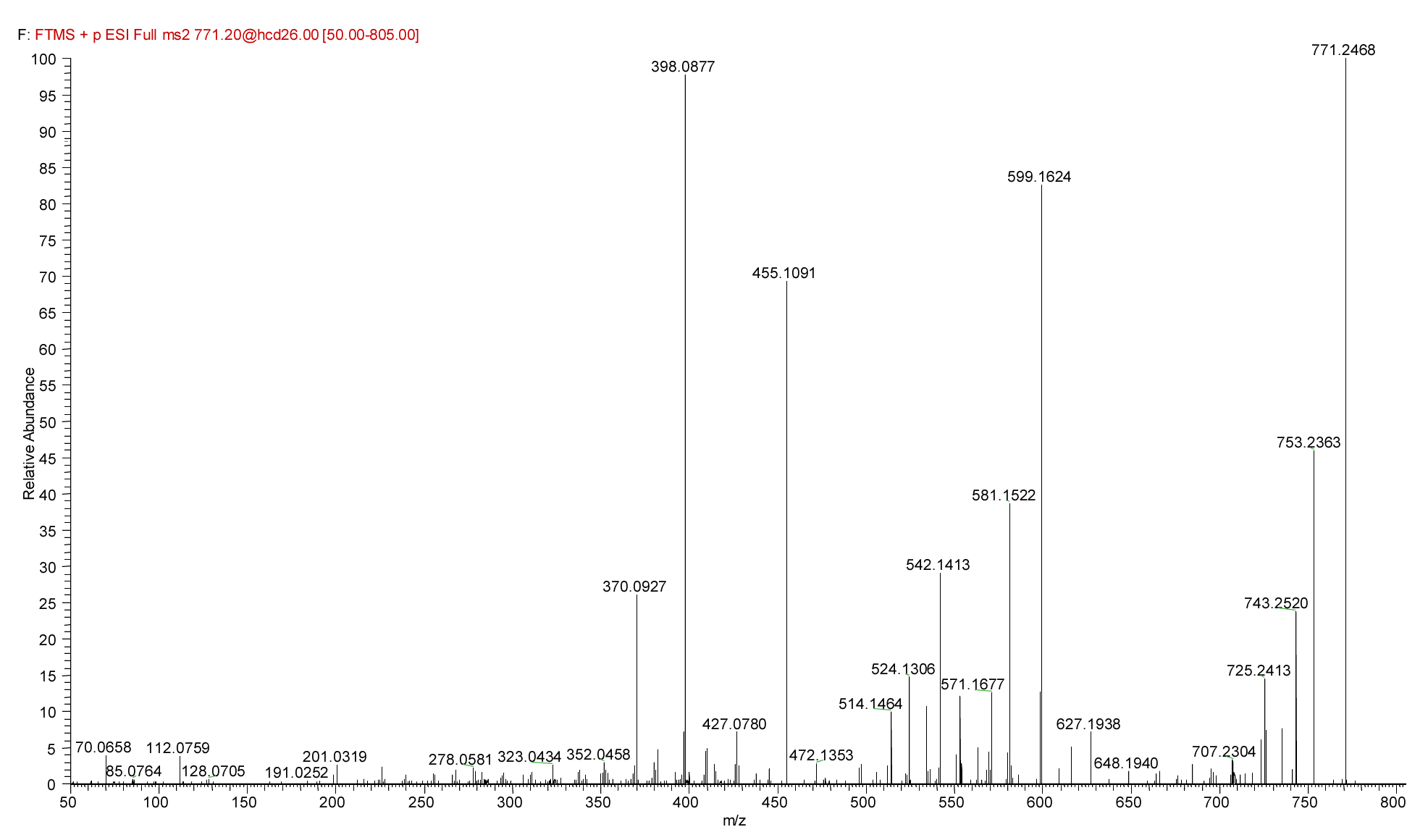

Supplement: S6 Fig — (TIF) [file pone.0150701.s006.tif]
