# Supplementary material for: HapX Mediates Iron Homeostasis in the Pathogenic Dermatophyte Arthroderma benhamiae but Is Dispensable for Virulence
Source: PLoS One. 2016 Mar 9;11(3):e0150701. doi: 10.1371/journal.pone.0150701 (PMC4784894; doi:10.1371/journal.pone.0150701)
Supplement: S1 Table — (PDF) [file pone.0150701.s007.pdf]

Primer	Sequence (5´-3´)[a]	Reference
AbenHAPX-1	CTGCTGGAAGGGCccGCGGAGGACCTG	This study
AbenHAPX-2	GAAGTTGACAagcTtATACTCCGTCTCTC	This study
AbenHAPX-3	GAAGATTTGCTGATggatccTAAAAGCTACG	This study
AbenHAPX-4	CAGATTTTTGGCgGCcgcGCTGATCATTAC	This study
AbenHAPX-5	ATATAAgATCTTTACTTAGAATCAGCAAATCTTCGATC	This study
AbenHAPX-9	CACCGGTAGATCggGCccAAAGATAGACAAG	This study

[a] The lowercase letters represent nucleotide exchanges introduced to create the underlined restriction sites.
