# Supplementary material for: HapX Mediates Iron Homeostasis in the Pathogenic Dermatophyte Arthroderma benhamiae but Is Dispensable for Virulence
Source: PLoS One. 2016 Mar 9;11(3):e0150701. doi: 10.1371/journal.pone.0150701 (PMC4784894; doi:10.1371/journal.pone.0150701)
Supplement: S2 Table — (PDF) [file pone.0150701.s008.pdf]

**Primer**	**Sequence (5´-3´)**	**Reference**
AbenACT-for	ATCTACGAAGGTTTCGCCCT	This study
AbenACT-rev	GCAGTCTGGATCTCCTCCTG	This study
AbenHAPX-for	CGTGCATCTACTCGTCAGGA	This study
AbenHAPX-rev	CACAGCTGAGCGTAATTGGA	This study
AbenSREA-for	GTCTGAGAATGGAGGACCCA	This study
AbenSREA-rev	CGTTTCTTAGTGTTGGGGGA	This study
AbenHEMA-for	TCTACCCCCAGCTACAATGG	This study
AbenHEMA-rev	TTGACTGCTCTGGTGTGGAG	This study
AbenCCCA-for	CCATACAACCTCCCAGAGGA	This study
AbenCCCA-rev	AGTATCCCAGTGCAAGGGTG	This study
AbenSIDA-for	TGAGCTCTTTAACCCCGAGA	This study
AbenSIDA-rev	CTTGGAGATCAGGCAGGAAG	This study
AbenSIDC-for	ATTGAGCCCGTAGTGTGGTC	This study
AbenSIDC-rev	ACCTTGATAGCGCTGATGCT	This study
AbenCYCA-for	TTCCTGCCACACCCTAGAAG	This study
AbenCYCA-rev	TCGGTGTAGGAGAAACCCTC	This study
AbenLYSF-for	TCGGCAATATTTTCTCCAGG	This study
AbenLYSF-rev	CTTCGTCGTACATCCCAGGT	This study
AbenHMG1-for	CCATGGCTACCACTGAAGGT	This study
AbenHMG1-rev	ACTGGCCCTCTTCAGAGTCA	This study
