# Supplementary material for: HapX Mediates Iron Homeostasis in the Pathogenic Dermatophyte Arthroderma benhamiae but Is Dispensable for Virulence
Source: PLoS One. 2016 Mar 9;11(3):e0150701. doi: 10.1371/journal.pone.0150701 (PMC4784894; doi:10.1371/journal.pone.0150701)
Supplement: S3 Table — (PDF) [file pone.0150701.s009.pdf]

Protein	Function	*A. fumigatus*	*A. benhamiae* homologue	Identity (%)
**Proteins involved in reductive iron assimilation (RIA)**				
FetC	ferroxidase	AFUA_5G03790	ARB_02127	58
FreB	ferric reductase	AFUA_1G17270	ARB_02049	45
FtrA	iron transporter	AFUA_5G03800	ARB_02126	56
**Proteins involved in siderophore biosynthesis**				
EstB	TAFC esterase	AFUA_3G03660	ARB_01286	28
PptA	phoshopantetheinyl transferase	AFUA_2G08590	ARB_04924	50
SidA	L-ornithine N^5-oxygenase	AFUA_2G07680	ARB_07687	60
SidC	ferricrocin NRPS	AFUA_1G17200	ARB_07686	27
SidD	fusarinine C NRPS	AFUA_3G03420	ARB_05131	38
SidF	hydroxyornithine transacylase	AFUA_3G03400	ARB_06708	48
SidG	fusarinine C acetyltransferase	AFUA_3G03650	ARB_05896	36
SidH	mevalonyl-CoA hydratase	AFUA_3G03410	ARB_00114	46
SidI	mevalonyl-CoA ligase	AFUA_1G17190	ARB_06663	45
SidL	GNAT-type acetyltransferase	AFUA_1G04450	ARB_01365	42
**Siderophore-iron transporters (SITs)**				
MirB	siderophore iron transporter	AFUA_3G03640	ARB_05150	28
MirC	siderophore transporter	AFUA_2G05730	ARB_03854	68
CccA	vacuolar iron importer	AFUA_4G12530	ARB_03795	66
**Regulatory proteins**				
HapX	bZIP transcription factor	AFUA_5G03920	ARB_06811	48
HapB	subunit of CBC	AFUA_2G14720	ARB_03649	74
HapC	subunit of CBC	AFUA_1G03840	ARB_01301	63
HapE	subunit of CBC	AFUA_6G05300	ARB_04269	77
SreA	GATA transcription factor	AFUA_5G11260	ARB_03037	38
SrbA	bHLH-LZ transcription factor	AFUA_2G01260	ARB_05762	45
PacC	C2H2 transcription factor	AFUA_3G11970	ARB_07827	54
MpkA	MAP kinase A	AFUA_4G13720	ARB_07820	81
AcuM	Zn cluster transcription factor	AFUA_2G12330	ARB_04776	61
